# Functional Roles of microRNAs in Agronomically Important Plants—Potential as Targets for Crop Improvement and Protection

**DOI:** 10.3389/fpls.2017.00378

**Published:** 2017-03-22

**Authors:** Arnaud T. Djami-Tchatchou, Neeti Sanan-Mishra, Khayalethu Ntushelo, Ian A. Dubery

**Affiliations:** ^1^Department of Agriculture and Animal Health, University of South Africa (Florida Campus)Pretoria, South Africa; ^2^Plant RNAi Biology Group, International Centre for Genetic Engineering and BiotechnologyNew Delhi, India; ^3^Department of Biochemistry, University of Johannesburg (Auckland Park Kingsway Campus)Johannesburg, South Africa

**Keywords:** agricultural crops, crop improvement, gene expression regulation, microRNA (miRNA)

## Abstract

MicroRNAs (miRNAs) are a class of small non-coding RNAs that have recently emerged as important regulators of gene expression, mainly through cleavage and/or translation inhibition of the target mRNAs during or after transcription. miRNAs play important roles by regulating a multitude of biological processes in plants which include maintenance of genome integrity, development, metabolism, and adaptive responses toward environmental stresses. The increasing population of the world and their food demands requires focused efforts for the improvement of crop plants to ensure sustainable food production. Manipulation of mRNA transcript abundance via miRNA control provides a unique strategy for modulating differential plant gene expression and miRNAs are thus emerging as the next generation targets for genetic engineering for improvement of the agronomic properties of crops. However, a deeper understanding of its potential and the mechanisms involved will facilitate the design of suitable strategies to obtain the desirable traits with minimum trade-offs in the modified crops. In this regard, this review highlights the diverse roles of conserved and newly identified miRNAs in various food and industrial crops and recent advances made in the uses of miRNAs to improve plants of agronomically importance so as to significantly enhance crop yields and increase tolerance to various environmental stress agents of biotic—or abiotic origin.

## Introduction

Plants form an important part of the ecosystem and may be used by humans as shelter, medicine and food. Major threats to plant productivity are human activities, abiotic stresses like drought, soil toxicity, climate change, and biotic threats like insects, herbivores, microbial pathogens, etc. The growth in human population with the concomitant increase in demand for plants and plant products warrants an increase in crop yields. One such method involves reducing the yield penalties by designing and adopting environmentally friendly crop protection measures. Advancement in molecular biology demonstrated the involvement of microRNAs (miRNAs) in regulating essential plant metabolic processes at the post-transcriptional level (Sanan-Mishra et al., 2009; Bej and Basak, 2014; Djami-Tchatchou and Dubery, 2015). These riboregulators can therefore serve as potential molecules that can be manipulated to help improve plant productivity. This review gives a brief description of miRNA biogenesis and discovery. It also discusses the role of miRNAs as potential gene regulators in cereals, legumes, tubers, fruits, biofuel sources, beverages, and fiber crops based on the most recent publications. The miRNA based strategies used for improving economically important plants are also described.

## Biogenesis of miRNA and their conservation in plants

**Figure 1:**
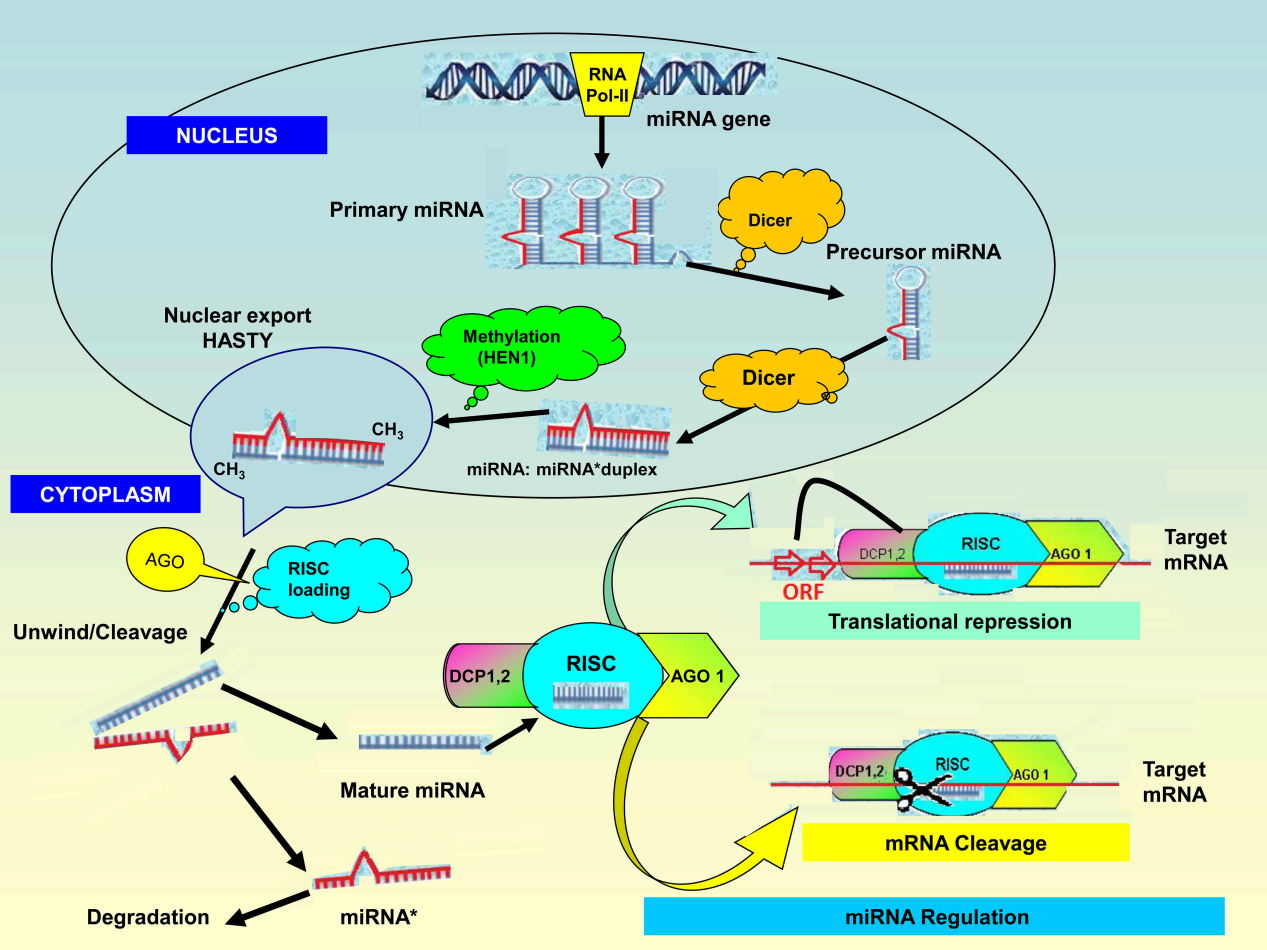
**microRNA biogenesis and mode of action**. The biogenesis of miRNAs starts in the nucleus where miRNA genes from distinct genomic loci are transcribed by RNA polymerase II into long primary transcripts (pri-miRNAs) followed by cleavage to precursor mRNA (pre-miRNA) by the nuclear RNase III-like enzyme, called DICER-LIKE (DCL1) in association with other proteins such as *hyponastic leaves 1* (HYL1) and *serrate* (SE). The imperfect stem-loop secondary structure of the pre-miRNA hairpins are cut by DCL1 enzymes to generate the miRNA:miRNA^*^ duplexes. The duplexes are methylated by *S*-adenosyl-L-methionine-dependent RNA methyltransferase, *HUA enhancer 1* (HEN1); and exported to the cytoplasm by HASTY where they undergo RNA-induced silencing complex (RISC) loading. The miR^*^ is released and the mature miRNA loads onto ARGONAUTE (AGO) ribonucleases in the RISC complex. Extensive base-pairing with mRNA targets is required for plant miRNA function to regulate gene expression. miRNAs guide Ago proteins to their specific targets through sequence complementarity which then leads to degrading the target mRNA transcript or by repressing its translation. On binding with perfect complementarity to a target mRNA, the Ago-miRNA complex induces its cleavage and degradation. An Ago-miRNA complex binding imperfectly to the 3′ UTR of the target mRNA induces translational inhibition, or deadenylation and subsequent decapping (DCP1,2) and degradation of the target mRNA.

miRNAs are non-coding RNA molecules ranging from 20 to 24 nucleotides in length (Jones-Rhoades et al., 2006). The miRNA genes (or *MIR* genes) are typically found in intergenic areas but can be also found in antisense—or sense orientation within introns of genes. Some miRNAs are clustered in the genome and are most likely to be transcribed together as long polycistronic RNAs. The biogenesis of miRNAs thus occurs in the nucleus (Figure 1) where specific *MIR* genes are transcribed by RNA polymerase II into long primary transcripts (pri-miRNAs; Bartel, 2004; Jones-Rhoades et al., 2006). These are typical transcripts capped at the 5′ end with a specially modified nucleotide and polyadenylated at the 3′ end with several adenosines (Pantaleo et al., 2010). The pri-miRNAs are then cleaved by RNaseIII-like enzymes called DICER-LIKE (DCL1) in association with other proteins such as *hyponastic leaves 1* (HYL1) and *serrate* (SE) into miRNA precursors (pre-miRNAs). These hairpin-looped structures are further processed by DCL1 to generate miRNA:miRNA^*^ duplexes in the nucleus rather than in the cytoplasm (Papp et al., 2003; Bartel, 2004; Peláez et al., 2012). The duplexes are methylated at their 3′ end by the conserved *S*-adenosyl-l-methionine-dependent RNA methyltransferase, *HUA enhancer 1* (HEN1; Sun et al., 2012) and exported to the cytoplasm by the plant homolog of EXPORTIN-5, HASTY (Park et al., 2005; Peláez et al., 2012). Here, the duplexes are loaded into the RNA-induced silencing complex (RISC), containing ARGONAUTE (AGO) proteins. After RISC loading, the miRNA:miRNA^*^ duplexes are unwound primarily by the AGO1 protein (Arribas-Hernández et al., 2016; Iki, 2017), and one strand of the duplex miRNA is directed to the exosome for degradation by a small-RNA degrading nuclease while the mature miRNA is inserted into the RISC which contains AGO proteins (Sun et al., 2012). Finally, the mature miRNA guides the RISC to complementary target mRNAs. miRNAs with high homology to the target mRNA lead to site-specific cleavage of the mRNA while miRNAs with imperfect base pairing to the target mRNA lead to translational repression and/or mRNA (Sun et al., 2012).

Several studies have shown that many miRNAs are evolutionarily conserved across all major lineages of plants, including gymnosperms, mosses, monocots, and eudicots (Cuperus et al., 2011; Sun, 2012). Based on the conservation and diversification of miRNAs during plant kingdom evolution, miRNA families can be grouped in two classes. The ancient miRNAs are often highly expressed and evolutionarily conserved, whereas the young miRNAs are expressed at comparatively low levels or only induced under specific conditions and generally exist only in few species, as a result being evolutionarily non-conserved (Qin et al., 2014). In Arabidopsis, evidence of frequent birth and death of *MIRNA* genes was previously reported (Fahlgren et al., 2007). The study gave an idea of new small RNA-generating loci having the potential to evolve into *MIRNA* genes through aberrant replication/recombination or transposition events from expressed gene sequences. Moreover, it suggested that many miRNAs are frequently lost during evolution (Fahlgren et al., 2007).

## miRNA-mediated regulation of gene expression

The diversity of these small RNAs is sufficient to specifically match nearly any given RNA encoded in a genome, thereby regulating almost every aspect of plant biology, often influencing control by transcription factors (TFs). The modes of action of miRNAs as important regulators of gene expression have been increasingly investigated (Sanan-Mishra and Mukherjee, 2007; Lelandais-Brière et al., 2010; Pantaleo et al., 2010; Sun, 2012; Djami-Tchatchou and Dubery, 2015). Most plant miRNAs are 20–24-nt in length and have been shown to regulate gene expression mainly in *trans* at the post-transcriptional level (Figure 1; Sun, 2012) through a high stringency of complementarity. It was later shown that perfect binding of a miRNA to the corresponding target mRNA results in the degradation of the target mRNA and imperfect pairing to the target mRNA leads to the translational repression (Sun, 2012). Cleavage initiates poly(A) tail removal leading to the destabilization of the target mRNAs and mRNA decay (Guleria et al., 2011). In addition to their role as post-transcriptional repressors of gene expression, miRNAs can help to confer robustness to biological processes by transcriptional silencing to reinforce transcriptional programs and to suppress random fluctuations in transcript copy number (Ebert and Sharp, 2012).

miRNAs of diverse lengths can be generated from different genes, as well as miRNAs that are heterogeneous in length from the same *MIRNA* gene (Starega-Roslin et al., 2011). Most plant pre-miRNAs are processed by DCL1 to generate 21 nt mature miRNAs, but DCLs 2–4 can also be involved to generate diverse lengths (Jeong, 2016). Such miRNA heterogeneity diversifies the miRNA pools and can potentially increase their regulatory potential. Moreover, this type of miRNA variation has practical implications for the construction of miRNA precursor-based expression casettes designed to release artificial miRNAs with specific sequences (Starega-Roslin et al., 2011).

## Molecular techniques adopted for miRNA research

### Approaches for miRNA isolation, identification, and characterization

The identification of miRNAs represents a critical and first step to elucidate their functions, and miRNAs have been primarily discovered by direct cloning and sequencing, genetic screening and bioinformatic predictions (Schommer et al., 2012). The first plant miRNAs such as miR156, miR159, miR164, miR171, etc., were described in *Arabidopsis thaliana* by isolating, cloning and sequencing small RNA populations (Llave et al., 2002; Park et al., 2002; Reinhart et al., 2002). By comparison, few miRNAs have been identified from genetic screens, possibly due to the lesser size of the small RNAs or redundancy with other miRNA-coding genes that have similar or identical sequences. However, the process of miRNA identification in Arabidopsis using the activation-tagging approach and genetic screening had proven to be powerful to isolate dominant miRNA mutants such as miR172a-2 (Aukerman and Sakai, 2003). Moreover, a genetic screen identified the *JAW* (jagged and wavy) locus, which produces a microRNA (miR-JAW) that can guide the cleavage of mRNAs of many *TCP* genes (TFs) controlling leaf development (Palatnik et al., 2003). Nucleotide mutations together with insertions/deletions can lead to the gain/loss of miRNA binding sites during co-evolution of miRNAs and their target genes (Guo et al., 2008) and screens for specific developmental defects caused by such mutations can assist in miRNA identification.

In the past decade, both computational strategies and experimental methods have been widely used to identify thousands of miRNAs in plants. Plant miRNAs exhibit a high degree of sequence complementarity to their target mRNAs and among the early computational approaches, homolog-based comparative genomic strategies predominated and conserved miRNAs were identified by BLAST (Basic Local Alignment Search Tool, www.ncbi.nlm.nih.gov/blast/) analysis of the known miRNA sequences against potential nucleic acid sequences [expressed sequence tags (ESTs), genome sequence surveys and genome sequences] of cotton (Zhang et al., 2007), switchgrass (Matts et al., 2010), wheat (Han et al., 2013), potato (Xie et al., 2011), and others.

The early experimental methods involved direct cloning and approaches based on genetic screening to identify and functionally analyze miRNAs in plants (Reinhart et al., 2002). Direct cloning was followed by Sanger sequencing and analysis and this has now evolved with the development of next generation deep sequencing (NGS) technology into a powerful tool for enhancing miRNA discovery and target identification in plants (Pantaleo et al., 2010; Kulcheski et al., 2011; Peláez et al., 2012; Djami-Tchatchou and Dubery, 2015). The identified miRNAs often need to be validated for expression by Northern blotting or by highly sensitive quantitative or semi-quantitative real-time PCR based assays (Shuzuo et al., 2012; Wang et al., 2012; Zhang and Wang, 2015).

In addition, techniques focused on the chromatography of proteins, mass spectrometry analysis, Western blotting, protein foot printing, etc., can be used for miRNA target identification at the protein level. For instance, a previous study using animal cells investigated the effects of miRNA regulation on the levels of many proteins by applying a quantitative mass spectrometry-based approach using SILAC (stable isotope labeling with amino acids in cell culture) and microarray data processing (Baek et al., 2008). Their results indicated that for most interactions miRNAs act as rheostats to make fine-scale adjustments to protein output.

The identification of miRNAs also led to development of databases containing searchable information on the miRNAs. The most useful tool for miRNA research is the miRBase (http://www.mirbase.org) which is a comprehensive and searchable miRNA database based on miRNA name, keyword, references, and annotation (Zhang and Wang, 2015). The Plant MicroRNA Database (PMRD; http://mirnablog.com/plant-microrna-database-goes-online/) contains a large amount of information for plant miRNAs, such as the miRNA and their target(s), secondary structure, expression profiling and genome browser (Zhang et al., 2010). The NGS analysis was also supported by complex computational programmes such as Next-Gen sequence databases (https://mpss.udel.edu/index.php), AthaMap (http://www.athamap.de/) (Steffens et al., 2004) and the CLC Genomic workbench 6 software (CLC Bio, Cambridge, MA, USA).

### Approaches for miRNA target prediction and target screening

Many of the target genes of plant miRNAs are predicted using bioinformatics tools followed by experimental approaches (Pantaleo et al., 2010; Zhang et al., 2015; Shriram et al., 2016). Some of the bioinformatics tools used for miRNA target prediction based on complementarity scoring and secondary structure analysis are: psRNATarget (http://plantgrn.noble.org/psRNATarget/) (Zhang and Wang, 2015); TAPIR (http://bioinformatics.psb.ugent.be/webtools/tapir/) (Zhang and Wang, 2015), miRTour (http://mirnablog.com/mirtour-plant-mirna-and-target-prediction-tool/; Zhang and Wang, 2015) and miRTarBase, the experimentally validated microRNA-target interactions database (http://mirtarbase.mbc.nctu.edu.tw/) (Hsu et al., 2011; Shriram et al., 2016).

While the expression levels of the target mRNAs can be monitored by real-time PCR, the target cutting site can be mapped by 5′-Rapid Amplification of cDNA Ends (RACE). Recently, the technique of degradome sequencing was developed. This is a modified version of 5′-RACE using a high-throughput, deep sequencing method (Song et al., 2011; Zhang B. et al., 2011; Zhang et al., 2015). The degradome sequencing approach also gives information on the relative abundance of cleaved targets (Zhang and Wang, 2015).

## Functional roles of miRNAs

Previous experimental studies in Arabidopsis and other plants have shown that miRNAs are involved in many biological processes where they play crucial role in development and growth, maintenance of genome integrity, signal transduction, hormone signaling pathways, hormone homeostasis, innate immunity, and response to different environmental abiotic and biotic stresses (Navarro et al., 2006; Sun, 2012; Xie et al., 2014; Zhang and Wang, 2015). Aspects of the regulatory roles of plant miRNAs during development, in the adaptive response to stresses and in the miRNA pathway itself have been reviewed (Mallory and Vaucheret, 2006) and more continues to be reported. For example, our recent topical study revealed that miRNAs can be utilized to reprogram cellular metabolism following the perception of microbe-associated molecular pattern (MAMP) molecules during pathogen attack in plants, leading to dynamic changes to the microtranscriptome associated with differential transcriptional regulation in support of immunity and basal resistance (Djami-Tchatchou and Dubery, 2015). Analysis of Tables 1–**3** shows the broad scope of miRNA effects, recurrently targeted at TFs, themselves controllers of gene expression. Another frequently occurring target is gene transcripts encoding proteins or enzymes involved in metabolism. In addition to summarizing the various functional roles ascribed to some conserved miRNAs in the different classes of crops in Tables 1–**3**, we discuss below the functions of newly identified miRNAs in crops.

**Table 1:** **The functional involvement of conserved miRNAs in cereal-, legume-, and tuber-crops**.

**Plant species**	**miRNA**	**Target genes**	**Functional involvement**	**References**
**CEREAL CROPS**				
Rice (*Oryza sativa*)	miR393	Auxin receptor gene (TIR1 and AFB2)	Drought response	Zhou L. et al., 2010
			High tillering and early flowering	Jiao et al., 2010
				Xia et al., 2012
	miR820	DRM2	Response to salt, high temperature	Sharma et al., 2015a
	miR167	ARF transcription factors	Cold-stress	Jeong et al., 2011
	miR397	L-ascorbate oxidase	Heat stress response and adaptation	Jeong et al., 2011
Maize (*Zea mays*)	miR156	Squamosa binding protein		Li et al., 2012
	miR160	ARF transcription factors	Development	Gu et al., 2013
	miR164	NAC1 (NAM, ATAF, CUC) - transcription factors	Endosperm development Ear development	Ding et al., 2013
	miR167	ARF transcription factors	Stress response	Sheng et al., 2015
	miR396	Growth factor		
	miR169	NF-YA transcription factors	Drought stress reponse	Sheng et al., 2015
Wheat (*Triticum aestivum*)	miR397, miR437	L-ascorbate oxidase	Development	
	miR395	ATP sulfurylase genes	Abiotic stress	Han et al., 2013
	miR1435/miR51812		Ion transportation	
Barley (*Hordeum vulgare*)	miR156d	Squamosa binding protein	Development, drought stress	Curaba et al., 2012
	miR396d	Growth factor	Seed development	Shuzuo et al., 2012
			Cell differentiation	
	miR399b	Phosphatase transporter	Drought stress response	
	miR164	ARF transcription factors	Lateral root and leaf development	Deng et al., 2015
**LEGUME CROPS**				
Soybean (*Glycine max*)	miR156, miR160	Squamosa binding protein		
	miR164, miR166	ARF transcription factors	Seed development	Song et al., 2011
	miR172, miR396	Growth factor		
Cowpea (*Vigna unguiculata*)	miR160 miR166		Drought stress	
	miR159 miR167	ARF transcription factors	Enhanced drought tolerance	Barrera-Figueroa et al., 2011
	miR169 miR319	Growth factor	Metabolic pathways of physiological changes associated with drought stress	Shui et al., 2013
	miR390, miR393			
	miR396, miR403			
	miR156b,f	Multicystatin gene	Protein degradation/ drought stress/ keep cellular proteins	Shui et al., 2013
Peanut (*Arachis hypogaea*)	miR156	Squamosa binding protein	Peanut growth and development	Chi et al., 2011
	miR159, miR171		Lipid and protein accumulation	Zhao et al., 2010
	miR159, miR396	Auxin response factors,		
	miR156, miR157	Lipid transfer protein, MYB TF, RLKs	Disease resistance	Zhao et al., 2015
	miR169, miR166			
**TUBER CROPS**				
Potato (*Solanum tuberosum*)	miR160	Auxin response factors	Growth and development	
	miR172		Starch accumulation	
	miR473	Serine/threonine kinase-like	Metabolism	Din et al., 2014
	miR475	Thioredoxin	Metabolism	
Sweet potato (*Ipomoea batatas*)	miR156, miR162	Squamosa binding protein transcription factors	Storage root initiation and development	
	miR167	ARF transcription factors	Stamen development	
	miR160, miR164, miR166, miR398	ARF, NAC1 transcription factors	Fibrous root and storage root development	Sun R. et al., 2015
Cassava (*Manihot esculenta*)	miR156, miR157, miR159, miR160	Transcription factors	Development stress response	Patanun et al., 2013
	miR164	NAC transcription factors	Drought tolerance	
	miR395, miR172, miR319, miR396 miR397	Transcription factors, Growth factor	Starch biosynthesis/metabolism	Chen et al., 2015
	miR414, miR473		Stress response	Patanun et al., 2013

### Functions of microRNAs in cereal crops

#### Rice (*oryza sativa*)

Rice is the primary source of food for more than half of the world's population. Like other crops, rice is challenged by various environmental stresses which include drought, salt, cold, heat, and nutrient deficiency which represent major limiting factors to its growth and yield. In 2004, 20 miRNAs were first identified in rice from a rice cDNA library and their predicted potential target genes were known to be involved in transport (miR-4, miR-15), disease resistance (miR-10), transcription (miR-4), metabolism (miR-5) etc. (Wang et al., 2004). The following year another study identified 35 rice miRNAs, of which 14 were novel and predicted to target genes involved in diverse physiological processes in rice (Sunkar et al., 2005). Today, many rice miRNAs have been identified with 592 miRNAs sequences present in miRbase version 21 (http://www.mirbase.org). Voluminous literature is present on the identification of miRNAs from rice NGS data and several conserved miRNAs are known to be involved in the response to abiotic stresses (Sunkar et al., 2007; Zhang, 2007; Mittal et al., 2016). Zhao et al. (2007) and Zhou M. et al. (2010) revealed that miR393 (*OsmiR393*), which targets two rice auxin receptor gene homologs (OsTIR1 and OsAFB2), was highly expressed under drought conditions. Its overexpression resulted in the reduction of tolerance to drought as well as salt (Xia et al., 2012). However, the *OsmiR393*-overexpressing plants showed two new phenotypes: high tillering and early flowering which are beneficial for productivity (Jiao et al., 2010). In other studies related to extreme temperatures (cold or heat), several cold-stress-responsive miRNAs (miR167, miR319, miR812q, and miR1425) were identified, which play an important role in modulating the expression of their target genes in response to cold conditions (Jeong et al., 2011). OsmiR397 has been reported as a high-temperature-responsive miRNA which modulates the expression of L-ascorbate oxidase (*OsLAC*) in response to the heat stress and adaptation of rice (Jeong et al., 2011; Yu et al., 2012). In addition it was reported that the overexpression of OsmiR397 and the downregulation of the corresponding *OsLAC* target gene resulted in the improvement of yield by increasing grain size and promoting panicle branching (Zhang et al., 2013). Another study revealed that the overexpression of the osa-miR7695 resulted in enhanced disease resistance to the fungal pathogen *Magnaporthe oryzae*, the cause of rice blast disease (Campo et al., 2013). Another recent study reported that Osa-miR820 plays a role in salt, high temperature, and drought stress responses (Sharma et al., 2015a).

#### Maize (*zea mays*)

Maize is the second most important food crop worldwide, used for food, feed, and forage and as a source of ethanol for fuel production. In addition to its agricultural and economic importance, maize has also been used in research as a model plant (Bennetzen and Hake, 2009). Five miRNAs including miR156, miR160, miR166, miR167, miR169 families involved in maize development, growth, and responses to biotic stress were firstly described and characterized with their potential target genes; squamosa promoter-binding protein, auxin response factor, HD-ZIP TF, CCAAT-binding factor, and HAP-2-like proteins, respectively (Mica et al., 2006).

miRbase version 21 lists 321 miRNAs of maize that play critical functions in growth, development and plant responses to biotic and abiotic stresses. It was shown that miR164 negatively regulates ZmNAC1 (NAM, ATAF, and CUC), a plant-specific TF family involved in development and stress regulation (Li et al., 2012). Moreover, 95 conserved miRNAs such as miR156a, miR160a, miR164e, miR164a, miR167d, miR168, miR169a, miR393a, miR397b, miR408b, miR528a, etc., belonging to 20 families, including 11 novel miRNA families, were identified and characterized to play regulatory roles during maize endosperm development (Gu et al., 2013). Another study showed that three maize miRNAs, miR528a (the regulator of a putative laccase, a Ring-H2 zinc finger protein and a MADS box-like protein); miR167a and miR160b (regulators of auxin response factor and a member of the B3 TF family), are important for ear germination, development and physiology of the plant (Ding et al., 2013). In addition to this finding, 72 genes (117 transcripts) targeted by 62 differentially expressed miRNAs such as miR159a, miR164a, miR171c, miR398a, miR408, miR528a, miR827, miRs7, miRs9, etc., which belong to 11 miRNA families, were identified and characterized to play important roles in ear development, providing a greater understanding of molecular mechanisms involved (Li et al., 2015). Recently, 124 conserved maize miRNAs and 68 novel maize miRNAs were identified to be associated with drought stress resistance where they play a crucial role in the regulation of genes involved in the drought stress response (Sheng et al., 2015).

#### Wheat (*triticum aestivum* L.)

Wheat is one of the most important cereal crops throughout the world due to its nutritional qualities and its massive contribution toward food security. There have been many efforts to investigate miRNAs at the subgenomic levels in wheat using sequencing which led to the discovery of 58 wheat miRNAs comprising 43 miRNA families; 20 of these families were conserved (miRNA156/157, miR159, miR160, miR164, miR165/166, miR167, miR168, miR169, miR170/171, miR172, miR319, miR390, miR393, miR396, miR397, miR399, and miR408,) and 23 were novel (miR501, miR502, miR 503, miR504, miR505 miR506, miR510, miR514, and miR516, etc.; Yao et al., 2007). They have been predicted to target genes such as: squamosa promoter binding proteins, MYB, NAC1, homeodomain-leucine zipper protein, auxin response factor, CCAAT-binding protein, scarecrow-like protein, APETELA2 protein, MADS box protein, and blue copper proteins involved in wheat development and diverse physiological processes (Yao et al., 2007). Many wheat miRNAs have been identified with 116 miRNAs sequences present in miRbase version 21. Sixty-two conserved miRNAs, belonging to 30 miRNA families and several new miRNAs were identified in wheat by EST analysis and their predicted potential target genes are known to be involved in various biological processes which include development (miR397, miR437), disease resistance (miR1436, miR1439, miR5067, miR5205), abiotic stress response (miR395d, miR1435), ion transportation (miR5181, miR5175), metabolic pathways (miR774, miR1126), and signal transduction (miR530, miR5175; Han et al., 2013). In a recent study, a genome wide survey of miRNAs was conducted on wheat tissues and 323 novel miRNAs belonging to 276 families were characterized with their associated target genes. These miRNAs were preferentially expressed in grain, suggesting that they play a crucial role in grain development (Sun et al., 2014). New abiotic stress-responsive miRNAs which include Ta-miR1122, Ta-miR1117, Ta-miR1134 and Ta-miR113, Ta-miR5653, Ta-miR855, Ta-miR819k, Ta-miR3708, and Ta-miR5156 were identified in wheat (Sun et al., 2014). These miRNAs regulate their corresponding target genes which encode BTB/POZ domain-containing proteins, F-box/Kelch-repeat proteins, ubiquitin carrier protein, as well as TFs involved in growth, metabolism, and stress responses (Pandey et al., 2013).

#### Barley (*hordeum vulgare* L.)

Barley is another important cultivated crop worldwide in terms of production. Barley grain is used for both human consumption and livestock feeding (Curaba et al., 2012). In addition to its agricultural importance, it is a model plant for the taxonomic family *Triticeae* and its genetics, genomics and breeding are well-studied (Sreenivasulu et al., 2008). About 100 miRNAs were identified for the first time in barley using deep sequencing of which 56 (miR156/157, miR159, miR319, miR160, miR172, miR393, miR396, miR408, etc.) had orthologs in wheat or rice that are known to be expressed, while up to 44 (miR437, miR444, miR528, miR2509) appear to be specifically expressed in barley. Similar to other important agricultural crops, barley has invoked interest for miRNA studies and 69 barley miRNAs are present in miRbase 21. In 2012, a combination of small RNA and mRNA degradome analyses enabled the identification and characterization of 84 conserved miRNAs (miR156, miR166, miR390, miR408, etc.) and seven novel miRNAs (hvu-miR5071b, hvu-miR1120b, hvu-miR6001, hvu-miR6002, hvu-miR6003, hvu-miR6004, and hvu-miR6005) together with 96 putative miRNA target genes predicted to be involved in various functions including carbohydrate translocation, cell differentiation, defense response, photosynthesis, and phytohormone signaling pathways (Shuzuo et al., 2012). This finding revealed that miRNAs play an important role in regulating early development of barley seed. They are involved in coordinating tissue specification and energy mobilization to ensure proper growth and development of the barley grain (Curaba et al., 2012). Using high-throughput Solexa sequencing of short RNAs, 133 novel, and 126 highly conserved barley miRNAs were identified from libraries (Shuzuo et al., 2012). The new miRNAs (miR-n026, miR-n028, and miR-n029), were suggested to play an important role in drought and salt stress responses. Deng et al. (2015) identified novel salinity responsive-miRNAs in barley and their corresponding target genes which improve their salinity tolerance.

### Legume crops

#### Soybean (*glycine max* L.)

Soybean is one of the most widely grown oil crop species in the world. It has a high content of seed protein (40%) and a high content of oil (20%) and is important for both human consumption and animal production. The interest of miRNAs in soybean began in 2008, when for the first time, 55 families of miRNAs of which 20 conserved (miR156, miR159, miR160, miR164, miR169, miR172, miR390, miR393, miR396, etc.) and 35 new miRNAs (miR1507, miR1509, miR1511, miR1512, miR1513, miR1515, miR482, etc.) were identified (Subramanian et al., 2008). Currently 639 soybean miRNA sequences are deposited in miRbase 21. Twenty-six miRNAs were identified to be involved in developing soybean seeds and their predicted targets included TFs from the MYB, ARF, NAC, GRF, and TCP-type gene families (Table 1), and also non-conserved genes, such as F-box protein, G-proteins, and protein suppressor of gene silencing 3 (Song et al., 2011). These findings revealed the regulatory network of miRNAs in soybean with their functions during seed development.

Like other plants, soybean is susceptible to environmental stresses such as drought, which is the major abiotic stress for soybean productivity around the world. Other studies focused on the regulation of soybean miRNAs in abiotic and biotic stresses. It was reported that small RNAs mediate gene regulation in roots under water deficit stress (Kulcheski et al., 2011). In wild soybean, a number of miRNAs were also detected to play important regulatory roles in Aluminum stress response (Qiao-Ying et al., 2012). Another important finding was the identification of miRNAs as key regulatory factors in response of soybean to devastating diseases which infect seedlings and mature plants like rust infection (Asian soybean rust; Kulcheski et al., 2011) and *Phytophthora sojae* root rot (Jing et al., 2011).

#### Cowpea (*vigna unguiculata* L.)

Cowpea is an economically important legume crop which grows in semi-arid and arid tropical regions in Africa, Asia, and Central and South America, where it is used as food for people and nutritious fodder for livestock. The ability of cowpea to adapt under drought conditions has made it an excellent system for studying the genetic basis of drought stress response. Work has been done to understand genetic elements, mechanisms of drought tolerance, and drought-associated miRNAs (Zhou L. et al., 2010; Barrera-Figueroa et al., 2011). A total of 47 miRNAs belonging to 13 miRNA families (miR156, miR160, miR172, miR319, miR399, miR2118, etc.) were first identified in Cowpea using computational approaches (Lu and Yang, 2010). About 30 potential target genes, encoding TFs, enzymes participating in regulation of development, growth, metabolism, and other physiological processes were subsequently predicted (Lu and Yang, 2010). Subsequently, numerous miRNAs have been identified in cowpea with 18 sequences deposited in miRbase 21 (Table 1). Some of these miRNAs were found to play important roles in drought tolerance (Barrera-Figueroa et al., 2011). The drought-associated miRNAs identified included miR156, miR160, miR162, miR164, miR166, miR159, miR167, miR169, miR171, miR319, miR390, miR393, miR396 miR403, miR482, and vun_cand030. miR169 enhanced drought tolerance by inducing the expression of nuclear factor Y TF orthologs (Barrera-Figueroa et al., 2011). The miRNAs are involved in regulating the expression levels of their corresponding drought-related target genes, to influence metabolism, development, and stress response (Shui et al., 2013). The Vun-miR5021 was predicted to target transcripts of cowpea responsiveness to dehydration (CPRD) –86 and –46, which are induced in response to water deficiency. CPRD46 encodes a neoxanthin cleavage enzyme involved in abscisic acid biosynthesis under water stress. The results indicated that vun-miR5021 can be considered as a miRNA marker for stress response in cowpea (Shui et al., 2013). The vun-miR156b and vun-miR156f were predicted to target the multicystatin gene involved in the regulation of the activity of cysteine proteinases which catalyzed protein degradation caused by drought stress. This regulation controls protein degradation rates during drought stress and keeps cellular proteins at the required level for plant survival.

#### Peanut (*arachis hypogaea* L.)

Peanut (also known as groundnut) is widely cultivated and is one of the five most important oil seed crops in the world. In 2010 the first miRNA-related research was performed on peanuts using high-throughput Solexa sequencing. Currently 14 novel miRNAs (miRn1 to miRn14) families (of these 13 are peanut-specific) and 75 conserved miRNAs (miR156 to miR894, etc.) which belong to 22 families are discovered (Zhao et al., 2010). The targets of 14 conserved miRNAs and seven new peanut miRNAs were predicted and include mRNAs for the GRAS (gibberellic-acid insensitive, repressor of gai, and scarecrow) family of TFs, nuclear TF Y subunit, NAC1 TF, protein synthesis proteins, auxin signaling F-box 3 protein, growth regulator factor 5, resveratrol synthase, basic blue copper protein, endonuclease, protein kinase, transport inhibitor response 1, and a disease resistance response protein (Zhao et al., 2010). The identified miRNAs were suggested to play an important role in growth, development and response to environmental stress. A year later another study identified 25 new peanut miRNAs and 126 conserved miRNAs which belong to 33 miRNA families (Chi et al., 2011). The target genes of the corresponding known identified miRNAs were involved in a large spectrum of biological processes and most of them were classified as TFs and functional proteins in plant metabolism and environmental stress response. The gene ontology analysis followed by the KEGG (Kyoto Encyclopedia of Genes and Genomes, http://www.genome.jp/kegg/) pathway of this study revealed that 34 miRNA families could be involved in 52 different biological processes such as carbohydrate metabolism, fatty acid biosynthesis, plant-pathogen interactions, response to stress, oxidation/reduction, and signal transduction (Chi et al., 2011). This finding also showed that miR156, miR159, miR171, and miR14 families had a total of four target genes involved in amino acid metabolism, fatty acid metabolism, and lipid metabolism; which suggested that miRNAs might have a crucial role in lipid and protein accumulation in peanuts. This study also showed that the target genes of the new miRNAs were involved in some specific developmental processes as they encoded the chlorophyll a/b-binding protein, heat shock protein, cold induced plasma membrane protein, aspartate carbamoyltransferase, and nitrate transporter and UDP-glucuronate 4-epimerase (Chi et al., 2011).

Recently, it was demonstrated that miRNAs play key roles in the responses of peanut to the widespread bacterial (*Ralstonia solanacearum*) wilt disease which reduces production significantly (Zhao et al., 2015). Upon bacterial wilt infection numerous miRNAs (miR3516, miR159, miR894, miR2199, miR1511, miR162, miR530, miR2118, miR396, miR3513, miR482, miR156/157, miR169, and miR166) were upregulated and some miRNAs (miR397, miR1508, miR4144, miR3515, miR408, miR2111, and miR3522) were downregulated. The target gene prediction of the differentially expressed miRNAs revealed that more than 10% of the predicted target genes were defense response genes which included: aquaporin, auxin response factors, GRAS family of TFs, hypersensitive-induced response protein, leucine-rich repeat (LRR) receptor-like serine/threonine-protein kinase, lipid transfer protein, MLP-like protein, and MYB TFs (Zhao et al., 2015).

### Tuber crops

#### Potato (*solanum tuberosum* L.)

Potato is the world's fourth most important food crop, following wheat, maize and rice. In 2009, 48 potential potato miRNAs (miR156, miR164, miR166, miR167, miR171, miR390, miR394, miR395, miR399, miR414, miR829, etc.) were first identified and predicted to target 186 genes involved in floral, leaf, root, and stem development, signal transduction, metabolism pathways and stress responses (Zhang et al., 2009).

Many conserved miRNAs were identified in potato through *in silico* approaches, and about 567 miRNAs are present in the miRbase 21 (www.mirbase.org) repository (Kim et al., 2011). One hundred and twenty potato miRNAs and their 433 targeted genes were identified to be linked to growth and development, metabolism, regulation, TFs, and physiological processes (Table 1). The novel miRNAs (stu-miR5254, stu-miR1450, stu-miR1879, stu-miR2673, and stu-miR5819) have been found to regulate TFs such as MYB, BZIP, bZIP21, homeobox-leucine zipper protein and the heat stress TF, HSFA9 (Din et al., 2014). Other novel miRNAs (stu-miR1446, stu-miR2090, stu-miR3951, stu-miR5150, stu-miR5205, stu-miR5449, and stu-miR6438), targeted an ABC transporter and enhanced potato tolerance to abiotic, biotic, and xenobiotic stresses (Din et al., 2014). In addition, some were also involved in disease-related proteins which include bacterial spot disease resistance protein 4, late blight resistance protein R3a and a TIR-NBS-LRR type disease resistance protein. Other identified miRNAs were found to regulate proteins involved in the process of cell signaling pathways such as a receptor-like protein kinase and a serine/threonine kinase-like protein (Din et al., 2014). The potato miRNAs such as miR172, miR477, miR1122, miR1127, miR1435, miR1438, miR1522, miR1533, and miR1535 play important roles in starch accumulation in tubers. They had a total of 14 targets, which were involved in carbon and sugar metabolism and sugar transport (Xie et al., 2011). It was reported that in solanaceous species such as potato, tobacco and tomato, NLR-targeting miRNAs have been identified and characterized as triggering abundant phased secondary small interfering RNAs (phasiRNAs), including the miRNAs miR482, miR5300, miR6019, and miR6027 (Fei et al., 2016).

#### Cassava (*manihot esculenta* crantz)

Cassava a perennial shrub, is widely grown as a staple food and animal feed. Together with maize, sugarcane, and rice, it represents a major source of energy for more than half a billion people in the tropical and subtropical regions of Africa, Asia, and Latin America (El-Sharkawy, 2004). In addition to its traditional importance, there is a growing demand for cassava starch for raw materials in a wide range of industries such as paper, textile, adhesive, and biofuels (Patanun et al., 2013). miRNAs have been identified from Cassava (Zhang et al., 2010). A number of conserved Cassava miRNAs and their target genes were identified which include miR156 (targeting squamosa promoter binding protein), miR159 (targeting Myb101), miR160 (targeting auxin response factor), miR162 (targeting Dicer-like1), miR168 (targeting AGO1), miR171 (targeting scarecrow-like TFs), miR390 (targeting protein kinase family members), miR394 (targeting F-box family protein), miR397 (targeting laccase 2), miR408 (targeting peptide chain release factor) etc. (Amiteye et al., 2011). One hundred and sixty nine potential cassava miRNAs which belong to 34 miRNA families, were identified using a computational approach (Table 1; Patanun et al., 2013) and about 306 miRNAs were released in miRbase 21. The newly identified cassava miRNAs also target various kinds of enzymes which include: sulfate adenylyltransferase, cinnamoyl-CoA reductase, laccase, multicopper oxidases, 5-methyl-tetrahydrofolate homocysteine methyltransferase, short chain alcohol dehydrogenase and acyl-protein thioesterase; known to play critical roles in various metabolic activities (Patanun et al., 2013). Nakashima et al. (2012), reported that 60 conserved miRNAs and 821 cassava-specific miRNAs contribute to drought tolerance. Another study reported that miRNAs are involved in defense against the bacterial pathogen *Xanthomonas axonopodis* pv. manihotis (Perez-Quintero et al., 2012). An interesting example of a stress-associated miRNA identified as having a potential role in the stress response in cassava, was miR164 which was predicted to regulate a NAC domain containing protein previously reported to be induced by drought and as well as conferring increased drought tolerance in plants (Nakashima et al., 2012). Recently, it was reported that a total of 107 conserved miRNAs (miR162, miR156, miR164, miR172, miR319, miR396, and miR397) which belong to 29 families and 39 new miRNAs (new-9, new-10, new-20, new-22, and new-26) which belong to 33 families, play crucial functions in plant development and starch biosynthesis/metabolism in cassava (Chen et al., 2015). Subsequently, Rogans and Rey (2016) also identified many conserved and new miRNAs with their predicted potential miRNA target genes known to be involved in important plant biological processes and regulation in cassava.

#### Sweet potato (*ipomoea batatas* L.)

Sweet potato, a perennial dicotyledonous plant which grows in temperate, subtropical and tropical areas across the globe is used for food as it contains carbohydrates, carotenoids, dietary fiber, minerals (calcium, potassium, and iron), vitamins (A, B, and C) and proteins. Sweet potato produces two types of roots, fibrous roots, and storage roots. The storage roots have high starch content with a sweet taste and are the portion of the plant consumed by humans. In addition to its use as staple food, sweet potato is used as a source of starch, animal feed, and also as a raw industrial material for alcohol production. Similar to other crops, numerous miRNAs have been discovered in sweet potato. The plant responds to wounding, by inducing the expression of miR828 which controls lignin and H_2_O_2_ accumulation by repressing the expression of *IbMYB* and *IbTLD* (Lin et al., 2012). The repression of *IbMYB* leads to the upregulation of the phenylpropanoid pathway toward the biosynthesis of lignin to strengthen the cell wall of a wounded plants. The repression of *IbTLD* expression led to the reduction of the expression of genes coding for antioxidant enzymes which results in a protection signal (Lin et al., 2012). Dehury et al. (2013) discovered conserved miRNAs in sweet potato by a comparative genomics-based approach using ESTs and genome survey sequences. This study identified eight potential miRNA candidates, which belong to the miR168 family (iba-miR1, iba-miR2, iba-miR4, iba-miR5, and iba-miR6); the miR2911 family (iba-miR3) and the miR156 family (iba-miR7 and iba-miR8). The target prediction revealed that the identified miRNAs in sweet potato regulate their target genes such as genes involved in transcription (targeted by iba-miR3 and iba-miR8), plant growth and development (targeted by iba-miR6, iba-miR7, and iba-miR8), signal transduction (targeted by iba-miR6 and iba-miR6), metabolism (targeted by iba-miR2, iba-miR5, iba-miR6, and iba-miR8), defense response (targeted by iba-miR5 and iba-miR6) and stress response (targeted by iba-miR2 and iba-miR5). Sweet potato miRNAs also play important regulatory roles during flowering, storage root initiation, and development (miR156 and miR162), fibrous—and storage root development (miR160, miR164, and miR166) and stamen development (miRNA167; Sun R. et al., 2015). This study also reported miR398 a stress-responsive miRNA.

### Fruit crops

#### Grapevine (*vitis vinifera* L.)

Grapevine is one of the most economically important and widely cultivated fruit crops in the world with good nutritional and processing properties. It is the first fruit crop to have its genome completely sequenced, attracting much interest from scientists for investigating miRNAs (Jaillon et al., 2007; Pantaleo et al., 2010). Because of the role played by miRNAs in regulating fruit development, a previous finding identified a total of 24 conserved miRNA families (example: miR156, miR160, miR172, miR395, miR408; Table 2), 26 known but non-conserved miRNAs (example: miR479, miR529, miR858, miR1432, miR1858) and 21 new grapevine-specific miRNAs (example: miRC1, miRC2, miRC3, miRC4 to miRC21; Pantaleo et al., 2010). Currently about 349 miRNAs of grapevine are deposited in miRbase 21. One hundred and twelve target genes of known miRNAs and 44 target genes of new grapevine-specific miRNAs were predicted for the better understanding of the functional importance of the identified miRNAs. The predicted target genes encode proteins involved in various biological processes such as fruit development, metabolism, photosynthesis, stress response, and TFs. In another study, 25 grapevine miRNA families were predicted to target a total of 134 potential target genes such as genes which encode TFs that are involved in plant growth, development, and phase change from vegetative to reproductive growth; target genes that encode enzymes such as ATP sulfurylase/APS kinase and ATP synthase which are involved in diverse metabolic processes; and target genes implicated in disease resistance, immune response, stress response, and signaling transduction (Wang et al., 2011). Since the key role of miRNAs in regulating fruit and seed development is of great applied interest for crop development (Han et al., 2014), identified a number of conserved and non-conserved grapevine miRNAs responsive to exogenous gibberellin and showed that they play a potential role in mediating gibberellin-induced regulation of berry growth, development and response to various environments. Another study identified six novel miRNAs expressed in berries during exogenous gibberellin application and/or ethylene treatment, supporting a role for miRNAs in grapevine fruit development and ripening (Wang et al., 2014). In line with the growing evidence that miRNAs play critical roles in both biotic and abiotic stress responses, a recent study reported that a diverse set of grapevine miRNAs are cold-inducible and may play an important role in cold stress response (Sun X. et al., 2015).

**Table 2:** **The functional involvement of conserved miRNAs in selected fruit- and biofuel-crops**.

**Plant species**	**miRNA**	**Target genes**	**Functional involvement**	**References**
**FRUIT CROPS**				
Grapevine (*Vitis vinifera*)	miR156	Squamosa binding protein	Fruit development	Pantaleo et al., 2010
	miR160, miR167	Auxin response factor	Development, stress response	Wang et al., 2011
				Han et al., 2014
	miR159, miR319	MYB transcriptionfactor	Phase change from vegetative to reproductive growth, stress response	
	miR393, miR394	F-box	Stress response	
	miR171, miR529	GRAS family transcription factors	Development, metabolism, photosynthesis	
Apple (*Malus domestica*)	miR156, miR159, miR166, miR167 miR172	Transcription factors	Plant growth and development, Stress response	Varkonyi-Gasicet et al., 2010
	miR169a, miR160e miR167b,g, miR168a,b	ARF transcription factors	Fire blight resistance	Kaja et al., 2015
	miR399		Phosphate homeostasis, Long distance signaling, Shoot to root transport	Pant et al., 2008
Orange (*Citrus sinensis*)	miR160	Auxin response factor 10	Root development, stress response	Song et al., 2012
	miR165	Homeo-domain leucine zipper and HD-Zip protein	Development, stress response, root absorption	
	miR172	AP2	Growth, Stress response	
	miR393	TIR1, ARF, and AFB	Adaptive responses of leaf to B-deficiency	Lu et al., 2014
	miR408	Cu homeostasis, superoxide dismutase	Tolerance to B-deficiency	Lu et al., 2015
Tomato (*Solanum lycopersicum*)	miR156/157	Colorless non-ripening	Fruit ripening	Xie et al., 2012
				Karlova et al., 2013
	miR172	APETALA2		
	miR319		Growth of leaf margins	Ori et al., 2007
	miR169	NF-YA transcription factors	Drought tolerance	Zhang X. et al., 2011
	miR390	RNA-induced transcriptional silencing complex protein TAS3	Leaf morphology	Karlova et al., 2013
	miR167, miR169, miR172, miR393, miR397	ARF transcription factors, NF-YA transcription factors	Cold/drought stress response	Koc et al., 2015
				Zhang X. et al., 2011
**BIOFUEL CROPS**				
Sugarcane (*Saccharum*)	miR156	SBP/SPL transcription factors	Development, stress response	Zanca et al., 2010
	miR159	MYB protein	Development	
	miR169	HAP12-CCAAT-box transcription factors	Salt stress tolerance	Carnavale-Bottino et al., 2013
	miR398	Serine/threonine kinase-like	Salt stress tolerance/ metabolism	
	miR164	NAC transcription factors	Drought stress response	Ferreira et al., 2012
	miR399	Inorganic pyrophosphatase 2	Drought stress response	
Switchgrass (*Panicum virgatum*)	miR156	SPL, Cg1gene, MYB, Heat shock protein-binding	Biomass production, Drought stress	Fu et al., 2012
				Shen et al., 2012
	miR167	ARF transcription factors, Glycosyl transferase-like protein	Biofuel yield, recalcitrance	Sun et al., 2012
	miR172	AP2, SPL3	Development, stress response	Sun et al., 2012
	miR159/319	MYB	Biofuel yield, development	Sun et al., 2012
	miR396	Growth-regulating factor	Drought and salinity stress	Xie et al., 2014
	miR397/408	Laccase		
	miR398	Fiber protein Fb2	Recalcitrance, abiotic stress	Sun et al., 2012
Sorghum (*Sorghum bicolor*)	miR156	SBP/SPL transcription factors	Development, increased biomass metabolism	Katiyar et al., 2012
	miR169	NFY	Development, drought response	Paterson et al., 2009
	miR398	Selenium binding protein	Transportation	Du et al., 2010
	miR170/171	GRAS domain transcription factors	Development	Zhang L. et al., 2011
				
	miR395	ATP, APS1 and Sultr1	Development, low Su response	Katiyar et al., 2012
	miR396	Growth-regulating factor	Development, stress response	
	miR397/398/408	Laccase	Response to Cu deficiency	
	miR399	UBC24 enzyme	Phosphate deficiency	Katiyar et al., 2012

#### Apple (*malus domestica* borkh)

Apple is cultivated worldwide as an economically important fruit tree, and its fruit is widely consumed. It is rich in flavonoids and other phenolic compounds, which may play a crucial role in reducing risk of chronic disease in humans (Boyer and Liu, 2004). Four hundred and thirteen miRNAs (miRBase 21) have been identified and characterized from apple, which help to understand their important regulatory mechanisms. A previous study showed that many miRNAs are expressed in the phloem tissue and phloem sap (Table 2; Varkonyi-Gasicet et al., 2010). This study demonstrated that some miRNAs in the phloem sap can play a long distance signaling role like a shoot-derived miR399 which is a long distance signal for the regulation of plant phosphate homeostasis with a shoot to root transport activity (Pant et al., 2008; Varkonyi-Gasicet et al., 2010). Xia et al. (2012) identified and characterized apple miRNAs, their expression patterns, targets and regulatory functions. It was revealed that miR159, miR828, and miR858 can collectively regulate up to 81 *MYB* TF genes potentially implicated in diverse aspects of plant growth and development. In another study using bioinformatic analysis and RNA library sequencing approaches, 146 miRNAs were identified from *M. domestica* (cv. Golden Delicious) of which 135 were conserved and 11 were novel miRNAs (Ma et al., 2014). This study showed that novel apple miRNA, Md-miRLn11, regulates the expression of a NBS–LRR protein during pathogen infection leading to resistance against the plant pathogenic bacterium (*Alternaria alternata* f.sp. mali; Ma et al., 2014). Recently, it was reported that four apple miRNAs (miR169a, miR160e, miR167b–g, and miR168a,b) are potentially involved in resistance to fire blight, a contagious bacterial disease caused by *Erwinia amylovora* by targeting stress response proteins (Kaja et al., 2015).

#### Orange (*citrus sinensis* L.)

The orange fruit can be eaten fresh, its juice which is known for its high vitamin C content is commonly consumed as a beverage and its peel has various uses from skin care to being an ingredient of household cleaning products. In addition to the economic value of the orange, availability of a large number of ESTs and the sequenced genome of citrus, have made it an excellent source of experimental material for elucidating the functions and the regulation pathways of miRNAs (Xu et al., 2013). Song et al. (2012) identified 15 potential *C. sinensis* miRNAs (csi-miRNAs) in leaves, stems, flowers, and fruits and predicted their corresponding target genes. About 124 orange miRNAs were identified in miRbase 21. The control of miRNA expression in orange root development can promote root growth and partially improve root absorption of more water and nutrients and also improve plant tolerance to abiotic and biotic stresses, resulting in increased plant biomass and yield (Song et al., 2012). The miRNAs were reported to play an important role in the adaptive responses of orange plants to nutrient deficiencies. Boron (B) deficiency is a widespread problem in *C. sinensis* and many other agricultural important crops. The study reported some miRNAs that regulate the adaptations of *C. sinensis* roots to B-deficiency (Lu et al., 2014) through several aspects which include the activation of the defense response, reactive oxygen species (ROS) signaling and scavenging due to upregulation of miR474 and downregulation of miR782 and miR843; the increase in the number of lateral roots by decreasing miR5023 expression and maintaining a certain phenotype favorable for B-deficiency-tolerance by enhancing miR394 expression; the increase of cell transport by decreasing the transcripts of miR830, miR5266, and miR3465; the improvement of osmoprotection by miR474 and the regulation of other metabolic reactions by miR5023 and miR821; finally the upregulation of miR472 and miR2118 expression in B-deficient roots leading to the disease resistance of roots (Lu et al., 2014). Most recently, Ma et al. (2016) reported on the miRNA regulatory mechanisms on orange leaves to magnesium (Mg)-deficiency, which affects crop productivity and quality. It was shown that the adaptive responses involved the downregulation of miR164, miR7812, miR5742, miR3946, and miR5158 that led to the induction of stress-related genes; the downregulation of miR158, miR5256, and miR3946 expression, leading to the activation of lipid metabolism-related genes; the downregulation of miR779, leading to the induction of cell wall-related gene expansin 8A and the repression of miR3946 and miR5158, resulting in upregulation of transport-related genes. In parallel, upregulated expression of miR395, miR1077, miR1160, and miR8019 and the induction of miR395 and miR6426, lead to the downregulation of the expression of genes involved in the maintenance of S, K, and Cu (Ma et al., 2016).

#### Tomato (*solanum lycopersicum* L.)

Tomato is one of the most popular and widely consumed vegetable crop and also a model plant for the study of ripening of fleshy fruit and senescence owing to its genetic and molecular tractability which attracted much interest from researchers for investigating miRNA function during fruit development by targeting genes involved in the ethylene pathway and fruit ripening (Giovannoni, 2007). Over 350–700 small RNAs from tomato fruit and leaf respectively were identified, with the majority reported to play an important role in fruit development (Itaya et al., 2008). miR1917, a key negative regulator of ethylene responses, was reported to play an essential role during tomato fruit ripening (Moxon et al., 2008). Subsequently it was found that the expression of many tomato miRNAs was tissue-specific and is involved in the regulation of fleshy fruit development (Moxon et al., 2008).

Knowing that in plants, miRNAs have been well-characterized and linked with stresses induced due to nutrient deficiency, Gu et al. (2010) demonstrated that miR398 in leaves, miR158 and miR837-3p in roots and miR399 in both roots and leaves are specifically induced by phosphate starvation in tomato.

In a recent experiment the role of miRNAs in tomato defense against *Fusarium oxysporum* was studied by demonstrating that miR482/2118 suppresses nucleotide-binding site (NBS) domain genes which confer resistance to *F. oxysporum* (Ouyang et al., 2014). Earlier it was also reported that miR159/319 and miR172 might contribute to viral pathogenesis in tomato against the Tomato leaf curl virus (ToLCV; Naqvi et al., 2010) Cold stress such as chilling and freezing, are among the most restraining abiotic factors that adversely affect tomato plant productivity. In a very recent study, Koc et al. (2015) identified and characterized some miRNAs which play an important role in tomato during cold stress response (Table 2).

### Biofuel crops

#### Sugarcane (*saccharum* sp.)

Sugarcane is an economically important crop from which ~75% of the global sugar is produced. It is becoming increasingly relevant in the production of renewable energy such as ethanol (Azevedo et al., 2011). Due to its biofuel importance, it has become a target for improvement of sustainable biomaterial production because of its high biomass productivity and built-in containment (Birch, 2007). Sugarcane has one of the most complex polyploid genomes not yet sequenced with publicly available ESTs and genomic survey sequence databases to assist in miRNA investigation (Jannoo et al., 2007). Many sugarcane miRNA precursors share high homology with their putative sorghum orthologs beyond miRNA mature sequence (Zanca et al., 2010). Moreover, there is high genome similarity between sugarcane and sorghum, making sorghum a good reference genome for analyses of sugarcane (Jannoo et al., 2007). Zanca et al. (2010), identified 19 sugarcane miRNAs with a total of 46 potential targets involved in various sugarcane biology processes.

A recent finding identified 98 conserved miRNAs which belong to 25 families, among which some were salt stress regulated miRNAs which play important roles in tolerance of sugarcane plants to salinity, such as miR528 that regulates a putative laccase (Table 2; Carnavale-Bottino et al., 2013). A recent study uncovered a complex regulation of sugarcane miRNAs in response to drought stress, one of the major constraints to sugarcane growth and development, and predicted miRNAs which target different TFs, proteins involved in tolerance to oxidative stress, cell modification, as well as hormone signaling. Some of these proteins were suggested to regulate sugarcane responses to drought, such as reduction of internode growth and shoot branching and increased leaf senescence (Gentile et al., 2015). It was shown using computational research that miR399 is also associated with drought stress response in sugarcane (Ferreira et al., 2012).

#### Switchgrass (*panicum virgatum* L.)

Switchgrass, a non-food crop, is a warm season perennial grass which originated from North America and a promising herbaceous lignocellulosic biofuel crop. The broad adaptation and rapid growth rate of switchgrass provide a stable and high supply of biomass for biofuel production (McLaughlin et al., 2006). Therefore, switchgrass has been the subject of increasing research including miRNAs investigation to improve biomass yield. Using bioinformatics approaches, 154 miRNAs have been identified in switchgrass, and their targets have been characterized (Matts et al., 2010). It was reported that the manipulation of the regulatory functions of miRNAs in switchgrass is a promising strategy to modify the lignin and cell wall phenolic ester synthesis to improve bioenergy production efficiency (Shen et al., 2012).

Studies demonstrated that salt stress has a gradual but significant negative impact on switchgrass growth and development. In turn, drought stress has little effect on the switchgrass germination rate (Sun et al., 2012). It was shown that miR156 and miR162 may play an important role in the adaption of switchgrass to drought stress and are good candidates for improving switchgrass as a biofuel crop by transgenic technology (Sun et al., 2012). Subsequently, Xie et al. (2014), identified miRNAs, together with their target genes, which may play important roles in switchgrass responses to drought and salinity stress (Table 2). In addition, at least 14 salt-responsive genes including a salt tolerance zinc finger protein, salt tolerance homolog 2, and low-temperature and salt-responsive protein family were identified to be miRNA targets. This study also demonstrated that certain miRNAs could be used to improve cellulose production by finding that at least 194 miRNA families regulated 449 target genes involved in carbohydrate and cellulose synthesis, which may contribute to biofuel production (Xie et al., 2014).

#### Sorghum [*sorghum bicolor* (L.) moench]

Sorghum is an important nutritious cereal crop that is highly resistant to drought, flooding, and external stress. Its uses include food, fodder and as a raw material for starch, alcohol, and biofuels production (Prasad et al., 2008; Mutegi et al., 2010). As a C4 plant species, it has high photosynthetic efficiency to convert solar energy to biomass and its high water use efficiency enables it to grow in areas prone to high temperature and drought as well as on poor and marginal lands. The potential agricultural use of sorghum and the growing demand of sorghum for biofuel production necessitate the development of high yielding cultivars with altered stem reserves, and improved resistance to pathogen and environmental stresses. In this regard, due to the roles of miRNAs in development, nutrient acquisition and use, and tolerance to abiotic and biotic stresses in plants, sorghum has also been an interest for miRNAs investigation (Katiyar et al., 2012). Currently about 446 sorghum miRNAs are available in miRbase 21.

Using a homology search based on the genomic survey sequence and the miRNA secondary structure, a previous finding identified 17 new miRNAs which belong to 11 miRNA families which can regulate 64 sorghum genes involved in cell cycle (tubulin folding cofactor B regulated by miR437), metabolism (NADPH-cytochrome P450 reductase regulated by miR1128), protein degradation (ERD1 protein regulated by miR827), RNA processing (exonuclease RRP41 regulated by miR1439), stress response (heat shock protein 70 regulated by miR528; serine/threonine protein kinase regulated by miR466), and transportation (selenium binding protein regulated by miR398; Du et al., 2010). A subsequent study identified 29 conserved miRNA families and 13 novel miRNAs using a sequencing approach to understand their post-transcriptional gene regulation (Zhang L. et al., 2011). The predicted targets for the novel miRNAs in sorghum were: E3 ubiquitin protein ligase regulated by miR5565; SNARE protein syntaxin regulated by miR5565d; SAM decarboxylase regulated by miR5570, putative receptor kinases regulated by miR5565f, and oxidoreductase/arabinogalactan protein regulated by miR5565e (Zhang L. et al., 2011). The plants' tolerance to drought and heat stress has been ascribed to the miR169 family as a probable reason for adaptation of sorghum to abiotic stresses (Paterson et al., 2009; El Sanousi et al., 2016; Hamzaa et al., 2016). These results indicated that in biofuel plants like sorghum, miRNAs may be a focus to improve biomass accumulation and the stress tolerance of the plant.

### Beverage crops

#### Coffee plants (*coffea arabica* L. and *coffea canephora* pierre ex froehner)

Coffee is one of the most important beverage crops and one of the major profitable agricultural commodities and is the second ranked on trade exchanges on the international community. Among the coffee plant species only *C. arabica* and *C. canephora* are economically important and responsible for 64 and 36% of the world production, respectively. Transcripts of *C. arabica* and *C. canephora* were sequenced and characterized and enabled miRNA investigation in these two species (Mondego et al., 2011). The first report on identifying miRNAs in coffee (*C. arabica*) was recently published (Akter et al., 2014). This study used a well-developed, powerful and comparative computational approach, and EST-based homology search for the first time and identified miR393 a large miRNA family with appropriate fold-back structures. The identified miR393 was predicted to target genes which encode a transport inhibitor-like protein, TFs, DNA-binding proteins, and a GRR1-like protein. These target genes play an important role in various biological processes like responses to chitin, cold, salt stress and water deprivation (Akter et al., 2014). Another subsequent study identified and characterized many new conserved and non-conserved miRNAs with their potential targets genes in both *C. arabica* and *C. canephora* (Loss-Morais et al., 2014). The targets included: medium chain reductase/dehydrogenases (MDR) family regulated by miR159e, photosystem I psaA/psaB protein regulated by miR393, serine/threonine-protein kinase-like protein regulated by miR479, Calcium-dependent kinase-like protein regulated by miR482a, probable LRR receptor-like serine/threonine-protein kinase regulated by miR482b, disease resistance protein RPM1 regulated by miR482c, F-box protein regulated by miR5225 and drought induced 19 protein regulated by miR8697 (Loss-Morais et al., 2014). The miRNA target genes predicted from the identified miRNAs in both coffee plant species revealed the roles of miRNAs in various cellular functions and gene regulation networks in these plant species. A most recent study using computational approaches identified 16 possible miRNAs in *C. arabica* and 20 in *C. canephora* all of which belong to 26 different families and 7 new miRNAs (car-MIR7122, car-MIR835, cca-MIR368, cca-MIR3169, cca-MIR5293, cca-MIR6459, and cca-MIR845). These identified coffea miRNA families regulated potential target genes involved in various biological processes and they strongly influence growth patterns, such as plant development, hormonal responses, mature mRNA formation, stress responses, and cellular signaling pathways (Chaves et al., 2015).

#### Tea plants [*camellia sinensis* (L.) kuntze]

The tea plant, source of an important commercial beverage crop of the world, is an evergreen woody perennial grown in different agro-climatic zones. The tea beverage possesses many health benefits to humans because its leaves contain polyphenols, catechins, caffeine, theanine, saponin, and volatile oils (Prabu and Mandal, 2010; Shi et al., 2011). In addition to its health benefits and economic value, the tea plant is of great interest for miRNA research due to the progress in functional genomics studies based on large-scale EST generation, analysis, and gene cloning. The first study on tea miRNAs, using computational methods, identified four candidate miRNAs which belong to four miRNA families with a total of 30 potential target genes (Prabu and Mandal, 2010). Of the four miRNAs identified, two belong to highly conserved miRNA families of miR164 and miR169, and the other two belong to specific miRNA families of miR1846 and miR1863. The 30 predicted target genes regulated by the newly identified miRNAs include six genes encoding TFs, six genes involved in cell development, seven genes involved in metabolic pathways (three genes for carbohydrate metabolism and four genes for protein metabolism), three genes involved in stress responses and eight hypothetical proteins (Prabu and Mandal, 2010). A subsequent study identified seven new miRNA families (miR408, miR414, miRf10132, miR2910, miR2914, miRf10185, and miR11590) which regulate genes encoding TFs such as MYB regulated by miR408; genes involved in proteosome degrading pathway such as ubiquitin conjugating enzyme, regulated by miR41; genes involved in nucleosome assembly such as histone H2B like protein regulated by miRf10132; genes involved in matrix organization such as extracellular matrix structural constituent, regulated by miR291; genes encoding glutamate metabolism related enzymes such as glutamate semialdehyde dehydrogenase, regulated by miR2914; genes involved in hydrolase activity such as carboxylic ester hydrolase, regulated by miRf10185 and genes involved in the regulation of flower development such as FRIGIDA protein, regulated by miR11590 (Das and Mondal, 2010). In line with these previous findings another study identified 14 new tea miRNAs which belong to nine families (Table 3), and predicted to potentially target 51 genes, which can act as TFs, and involve in stress responses, transmembrane transport and signal transduction (Zhu and Luo, 2013). Cold stress negatively affects the growth, development, and spatial distribution of the tea plant by causing tissue injury and growth delay, which decrease its yield and quality. A recent study identified and characterized cold-responsive miRNAs in tea plant indicating that miRNAs play a crucial role in tea response to cold stress (Zhang et al., 2014).

**Table 3:** **The functional involvement of conserved miRNAs in selected beverage-, fiber-, and other-crops**.

**Plant species**	**miRNA**	**Target genes**	**Functional involvement**	**References**
**BEVERAGE CROPS**				
Coffee (*Coffea arabica* and *Coffea canephora*)	miR167	Auxin response factor	Development, stress response	Chaves et al., 2015
	miR159e	Medium chain reductase/dehydrogenases	Development, stress response	Loss-Morais et al., 2014
	miR393	Transport inhibitor-like protein, DNA-binding proteins, GRR1-like protein	Chitin, cold, salt stress, and water deprivation	Akter et al., 2014
	miR171	GRAS family transcription factors	Development, metabolism	Chaves et al., 2015
	miR390	TAS3	Development, cellular signaling pathways	
Tea plant (*Camellia sinensis*)	miR156	SBP/SPL transcription factors	Plant growth, development	Zhu and Luo, 2013
			Cold Stress response	
	miR171	GRAS family transcription factors	Development, stress response	
	miR397	Laccase	Stress responses	
	miR399	Ubiquitin-conjugating enzyme	Stress responses	
	miR408	Plastocyanin-like	Cold stress	Zhang et al., 2014
**NON-FOOD CROPS**				
Cotton (*Gossypium hirsutum*)	miR156	SBP/SPL transcription factors	Development, stress response	Wang and Zhang, 2015
	miR172	AP2, SPL3	Flower development, phase change	
	miR319	MYB protein	Controlled leaf development	
	miR396	Callose synthase	Cotton fiber development	Zhang et al., 2007
	miR167a	ARF transcription factors	Salt stress tolerance	Yin et al., 2012
	miR395	APS1		Wang et al., 2013
	miR397a/b	Laccase		
	miR399a	UBC24 enzyme		
Tobacco (*Nicotiana tabacum*)	miR156	SPL	Development, stress response	Guo et al., 2011
	miR160/167	ARF transcription factors	Development, stress response	Frazier et al., 2010
	miR164	NAC transcription factors	Lateral root development	
	miR169	NFY	Development, drought	
	miR171	GRAS domain transcription factors	Development, growth	
	miR172	AP2, SPL3	Development, stress response	
	MiR319	MYB protein	Development	
	miR393	ARF and AFB	Development, stress response	
	miR166	Leucine-rich (LRR) repeat family	Disease resistance	Guo et al., 2011
	miR399	4-Coumarate-coenzyme A ligase	Stress response	
	miR408		Response to wounding and topping	Tang et al., 2012

### Non-food crops

#### Cotton (*gossypium hirsutum* L.)

Cotton is among the most important economic crops due to its role in providing natural textile, fiber, and edible oil. In addition to the fact that cotton is the most important fiber crop, it is also the second most important resource of plant protein, the fifth most important oil crop and is a model species for investigating plant polyploidization and cell wall and cellulose biosynthesis (Wang et al., 2012). Following the first reports on identification of miRNAs in cotton in 2007, hundreds of miRNAs (158 in miRbase 21) have been identified and characterized (Zhang et al., 2007; Barozai et al., 2008). These studies revealed that cotton miRNAs are associated with many biological and metabolic processes which include fiber initiation and development, floral development, embryogenesis, and responses to pathogen and environmental stresses (Zhang et al., 2007; Abdurakhmonov et al., 2008). The findings of Zhang et al. (2007), demonstrated that miR396, miR414, and miR782 regulated callose synthase, fiber protein Fb23 and fiber quinone-oxidoreductase and indicated the important roles of miRNAs in cotton fiber differentiation and development.

The role of miRNAs in response to salinity stress was investigated and found that 17 cotton miRNAs which belong to eight families were identified and it was found that more miRNAs responded to salinity treatment in a salt-tolerant cultivar than in a salt-susceptible cultivar (Yin et al., 2012). Recent evidence highlighted that miRNAs also play a role during cotton's heavy metal-related stress responses that include changes in chloroplast ultrastructure, inhibition of growth, and photosynthesis and induction of cell membrane damage (He et al., 2014). Treatment of cotton with lead significantly altered the expression pattern of miR159, miR162, miR167, miR395, miR396, miR156, miR398, miR399, miR414, miR833a, and miR5658 and their targets in both leaves and roots at a dose- and tissue-dependent manner. Various studies reported on cotton miRNAs responding to biotic stress and suggest miRNA regulation of cotton's defense against insect and disease invasion. One example is that of a recent study in which about 140 miRNA families and 58 new miRNAs and their potential targets were shown to be involved in the regulation of cotton defense responses against Verticillium wilt, a vascular disease which significantly affects cotton growth and development (Zhang et al., 2015).

#### Tobacco plant (*nicotiana tabacum*)

Tobacco is an important economic and agricultural non-food crop in the world which has also been investigated as a potential biofuel crop (Andrianov et al., 2010). It is a model plant for studying fundamental biological processes and alkaloid secondary metabolites. It is also the source of the plant cell line, which has been used as key material for plant molecular research. Recently the draft genome of tobacco has been released (Sierro et al., 2014), in addition to the Genome Survey Sequences (GSS) sequences deposited in the public database which enabled some transcriptomic and genomic studies as well as miRNAs studies. In a first attempt Frazier et al. (2010) used computational approaches to identify 259 potential tobacco miRNAs which belong to 65 families and predicted 1225 potential tobacco miRNA target genes which included TFs, DNA replication proteins and metabolic enzymes which play an important role in tobacco growth and development.

Topping (the removal of the flowering head and young leaves of the tobacco plant) is an important cultivating measure for flue-cured tobacco which switches the plant from reproductive to vegetative phase. It is used to increase tobacco leaf production and to encourage leaf ripening. Since many genes had been found to be differentially expressed in response to topping, it had been proved from a previous study that miRNAs (136 conserved miRNAs belonging to 32 families and 126 new miRNAs belonging to 77 families) were differentially expressed in flue-cured tobacco roots before and after topping and regulated transcripts distinctly involved in response to topping (Guo et al., 2011). They found that 15 miRNA families such as miR156f, miR171d were involved in development, which is consistent with the transition from reproductive to vegetative phase induced by topping. Since topping is considered as wounding stress, they found that seven miRNA families were involved which included nta-miRn70 which targets UDP-glucosyl transferase family protein which plays important roles in stress responses of plants by glycosylating hormones and secondary metabolites; nta-miRn11b which targets a disease resistance protein and nta-miR166c which targets a LRR family protein which has functions in disease resistance. They also found that topping can promote roots to take up nitrate by repressing nta-miRn73 which targets a nitrate transporter (Guo et al., 2011). Moreover, nta-miR164a targets the NAC1 TF which mediates auxin signaling to promote lateral root development (Guo et al., 2011). In addition, another study experimentally confirmed the expression of known miRNAs and their changes upon wounding or topping treatment and identified novel tobacco-specific miRNAs using a high-throughput sequencing approach. This study detected the expression of 100 known miRNAs which belong to 27 families in roots and leaves which include: miR408, miR477, miR1919, miR2118, and miR2911 and 59 novel tobacco-specific miRNAs which belong to 38 families. In this study the identification of wounding- and topping-responsive miRNAs and defense-related targets of these small RNAs indicated that the inducible defense responses in tobacco are controlled by pathways involving miRNAs (Tang et al., 2012).

A more recent study demonstrated that miRNAs regulates nicotine biosynthesis in tobacco by identifying four unique tobacco-specific miRNAs which were predicted to target key genes of the nicotine biosynthesis and catabolism pathways and an endogenous target mimicry, novel tobacco miRNA eTMX27 (miRX27) which targets quinolinate phosphoribosyltransferase-2 encoding a quinolinate phosphoribosyltransferase (Li et al., 2015).

## miRNA-based strategies for improving plant crops

Functional analysis of miRNAs demonstrated their importance in multiple biological and metabolic processes in economically important crops. Many studies reported that miRNAs are among the most important gene regulators (riboregulators) which control plant growth, development and response to abiotic and biotic stress in plants. Therefore, miRNA-based genetic modification technology is one of the most promising solutions which can contribute to agricultural productivity in order to produce superior crop cultivars.

Transgenic approaches toward manipulating the miRNA-based control on gene expression include the overexpression of miRNA-resistant targets as well as the creation of artificial target mimics (a newly developed technology used to repress the activity of specific miRNAs; Gupta, 2015). This proves to be an excellent crop improvement strategy for stress tolerance when the miRNAs of interest operate as negative stress regulators, in which transgenic plants overexpressing these miRNAs are susceptible to stresses (Franco-Zorrilla et al., 2007). Another possible approach is the use of artificial miRNAs (amiRNAs) designed to suppress target gene expression of a protein-coding mRNA of interest. This is a novel posttranscriptional gene silencing approach which has been used efficiently in rice and several plant species from dicots to moss (Khraiwesh et al., 2008; Sharma et al., 2015b). The artificial miRNAs technology was used to target the cucumber mosaic virus suppressor 2b. By efficiently inhibiting 2b gene expression it conferred resistance to transgenic tobacco plants to this virus (Qu et al., 2007). This approach can be used to create transgenic plants for improving crop tolerance to abiotic and biotic stresses and for increasing yield.

### MicroRNA-based strategies to improve crop tolerance to abiotic and biotic stresses

The plant molecular responses to abiotic stresses involve interactions and crosstalk with many molecular pathways including miRNA regulation (Bej and Basak, 2014). Therefore, breeding new crop plant cultivars with improved tolerance to abiotic stress is important for maximizing crop yield and quality. A study demonstrated that in tomato plants, the over-expression of a drought responsive miR169 enhanced drought tolerance by lowering stomatal opening, which decreased transpiration rates and reduced leaf water loss (Zhang X. et al., 2011). In rice it was demonstrated that the over-expression of osa-MIR396c led to a susceptible plant by reducing salt and alkali stress tolerance (Gao et al., 2010). Similarly, transgenic rice lines which overexpressed the miR398-resistant form of rice Cu- or Zn-superoxide dismutases showed more tolerance to high salinity and water stress than non-transgenic rice (Lu et al., 2011). The over-expression of miR319 impacted on leaf morphogenesis and led to enhanced cold tolerance after chilling acclimation in rice (Yang et al., 2013).

Recent advancements in plant miRNA research have also demonstrated their functions in crop responses to biotic stresses. It was shown that the over-expression of Osa-miR7696 confers resistance to blast infection in rice (Campo et al., 2013). Similarly, the overexpression of miR393 significantly inhibits the growth of bacteria providing a tool for plant immunity to disease (Navarro et al., 2006). Another study demonstrated that the overexpression of *miR160a* positively regulates MAMP-induced callose deposition, while *miR398b* and *miR773* negatively regulate MAMP-induced callose deposition and resistance to bacterial infection. This indicates the potential of further research into the role of miRNA regulation in plant innate immunity (Li et al., 2010).

Many studies reported on the functional analysis of miRNAs in a model plant such as Arabidopsis (Zhang and Wang, 2015), but limited translational investigations were done on the functional analysis of key miRNAs in important crop plants. Therefore, from the knowledge that miRNAs play key roles during plant response to stress, it is anticipated that regulation of their expression in specific crop plants of interest can greatly improve tolerance to abiotic and biotic stresses.

### microRNA-based strategies to improve crop growth and development

Various previous studies demonstrated that miRNAs play an important role in plant growth and development (e.g., leaf development, apical dominance, and plant biomass). Thus, development of transgenic plants which express miRNAs associated with these physiological processes is growing as a new approach for improving plant architecture, grain yield, fruit improvement and enhanced shelf life. In tomato overexpression of miR319 resulted in larger leaflets and continuous growth of leaf margins (Ori et al., 2007). Previous evidence from model plants showed that the overexpression of miR156 results in the increase of the shape and the number of plant leaves which can be 10 times higher than in normal wild-type Arabidopsis (Schwab et al., 2005). Recent evidence showed that the overexpression of miR156 in switchgrass inhibited plant apical dominance which led to the increase in tiller numbers and an increase in biomass yield of 58–100% in transgenic plants (Fu et al., 2012). Similarly, in tomato the overexpression of miR156 decreased apical dominance, increased the branching of plants and leaves, and further resulted in high plant biomass (Xie et al., 2012). In rice it was shown that the overexpression of microRNA OsmiR397 improved rice yield by increasing grain size and promoting panicle branching (Zhang et al., 2013). In addition, the overexpression of miR319 also caused an increased number of longitudinal small veins in rice (Yang et al., 2013). miR390 plays an important role in enhancing lateral root development (Marin et al., 2010; Yoon et al., 2010) by negatively regulating the expression of repressors for lateral root growth ARF2, ARF3, and ARF4 (Marin et al., 2010). In *Medicago truncatula*, a model legume for legume biology, it was demonstrated that the overexpression of miR160, one of the most important gene regulators for root development and gravitropism, showed distinct defects in root growth and nitrogen-fixing nodule formation, including decreased root length and severe disorganization of the root apical meristem (Bustos-Sanmamed et al., 2013). These findings can be extrapolated to legume crops with economic importance such as soybean, cowpea, and peanut for their growth and development improvement. It has also been well-documented that miRNAs also play an important role in regulating floral development and vegetative phase change in Arabidopsis, rice and maize. In maize, the APETALA-like gene *glossy15* regulates the phase change from vegetative growth to reproductive growth including the transition from a juvenile to adult leaf (Lauter et al., 2005). A study demonstrated that the overexpression of miR172 lead to the repression of the expression of *glossy15* which resulted in delayed phase change from vegetative to reproductive in maize (Lauter et al., 2005). Additionally, Chuck et al. (2007) speculated that high miR156 and low miR172 expression promote juvenility, whereas high miR156 and miR172 expression promote the adult reproductive phase in maize (Chuck et al., 2007).

Since many functions of miRNAs are investigated by overexpression or lowered expression of miRNAs in the plant of interest, the manipulation of miRNA expression levels can be conducted following this pattern to confirm miRNA functions in important plant crops and can provide an effective strategy for improving plant development, fruit, and seed development as well as plant biomass yield.

### Genome editing technologies for miRNA manipulation

Given the functionally diverse roles of miRNAs in plant crops, a number of studies have been using the traditional overexpression and repression strategies to investigate the function of miRNAs (Lauter et al., 2005; Navarro et al., 2006; Chuck et al., 2007; Gao et al., 2010; Zhang X. et al., 2011; Campo et al., 2013). However, due to the short length of miRNAs, the effectiveness of the developed strategies for down-regulation of miRNA or miRNA loss-of-function are relatively less robust. However, two genome editing technologies with engineered nucleases have become powerful tools for targeted modifications to the genome, providing unprecedented control for specific genome engineering. These are based on the creation of double-stranded breaks at target sequences by TALENs (Transcription Activator-Like Effector Nucleases) and CRISPR (Clustered, Regularly Interspaced Short Palindromic Repeats) for gene modification, deletion or addition (reviewed by Khan et al., 2017). Especially the CRISPR-Cas9 technology has emerged as a novel (and non-transgenic) RNA-guided genome editing and targeted gene mutation tool due to its simple structure and its applicability to a variety of organisms (Chan et al., 2016; Schiml and Puchta, 2016). A previous study using rice demonstrated that Cas9 could be guided by engineered gRNA for precise cleavage and editing of the plant genome (Xie and Yang, 2013). Another study showed that the CRISPR/Cas9 system is effective in soybean by knocking-out a green fluorescent protein transgene and modifying nine endogenous loci (Jacobs et al., 2015). In a very recent study using a human cancer cell line, CRISPR/Cas9 constructs was cloned with single-guide RNAs specifically targeting biogenesis processing sites of selected miRNAs (miR-17, miR-200c, and miR-141). This proof of concept study showed that CRISPR/Cas9 can robustly and specifically reduce the expression of these miRNAs up to 96% (Chan et al., 2016). The application of CRISPR-cas9 technology has revolutionized gene manipulation capabilities and has been successful in model plants such as tobacco and *Arabidopsis*, and crops plant such as wheat, maize, rice, sorghum, tomato, and sweet orange, but its application in editing non-coding RNAs in plants is still nascent (Bassak and Nithin, 2015). Due to the emerging promise of the CRISPR/Cas technology, it can be also exploited as a powerful genome-editing and gene-targeting tool for functional genomic characterization of plant miRNAs/genes and genetic modification to improve of agricultural crops.

## Conclusion and future perspectives

As exemplified by the case studies discussed, miRNAs are currently regarded among the most important gene regulators. In the past few years, significant progress has been made to analyze and characterize plant miRNAs, with an increasing number of research reports on the crucial function of miRNAs in crop plants. This review summarizes findings regarding plant miRNAs and the versatile functions in agronomically/economically important plants. As discussed, these small nucleic acids are involved in vital aspects which include plant growth and development, vegetative to reproductive phase change, hormone signaling, and signal transduction pathways and homeostasis responses to different environmental abiotic and biotic stresses (Sun et al., 2012). Many recent studies also revealed that plant miRNAs are involved as molecular regulators of plant immune and defense responses (Li et al., 2010; Balmer and Mauch-Mani, 2013; Djami-Tchatchou and Dubery, 2015). With this increasing evidence of the importance in crop plants, miRNAs are thus emerging as the next generation targets for genetic engineering for improvement of the agronomic properties of crops.

In this regard, the manipulation of miRNA expression levels would represent an effective strategy for improving the responses of crop plants to environmental stress, attack by pathogens and parasites as well as plant growth and development (Zhang and Wang, 2015). In practice, different transgenic approaches, focused on miRNAs of importance and the corresponding identified target genes, can be used. These include constitutive overexpression of miRNAs, stress-induced, or tissue-specific expression of miRNAs or the targets, expression of miRNA-resistant target genes, artificial target mimics and artificial miRNAs (Zhou and Luo, 2013; Gupta, 2015).

In cases where the native target gene has an undesirable effect, the constitutive overexpression of the regulating miRNA results in suppression of the corresponding mRNA, a strategy that may be used for crop improvement when the miRNAs of interest operate as positive stress regulators (Yang et al., 2013). Where the target gene has a desirable effect on the trait of interest (with the miRNA serving as a negative regulator), strategies followed can include overexpression of the target gene or selection of miRNA-resistant target genes or artificial target mimics (Gupta, 2015).

Although successful in general, the practical agricultural application of these plant miRNA methods is challenging since the engineering of complex multi-genic traits such as yield may require tuning of expression of several genes during various phases of plant development. However, detailed knowledge of miRNA-based target regulation in model plants and crops would further benefit the design and strategy for improving these above-mentioned methodologies for crop improvement. Another obstacle, as mentioned previously, is that miRNA overexpression and knockout of major target genes normally produce very similar phenotypes, and this is generally contrary to what is observed in plants with reduced activity of the miRNA (Palatnik et al., 2007). It is also possible that some miRNAs affect target expression only in specific cell types, and solely under particular conditions as many miRNAs and the targets may have intricate expression patterns. In this case, expression analysis in whole organs would obscure the effects of miRNA; therefore the approach should be carefully and specifically designed for better results.

The use of artificial miRNAs designed to suppress target gene expression of a protein-coding mRNA of interest is regarded to be among the valuable approaches for crop improvement applications. However, the effects of many important miRNAs in living cells may not be visible due to inadequate concentration or expression. In this case it would be necessary to perform quantitative analysis of the natural plant miRNA(s) present in cells, as well as that needed for visible effects, before designing the artificial miRNAs directed toward obtaining plants with desirable agronomical traits. Although this artificial miRNA method is already used as an effective tool (Khraiwesh et al., 2008; Sharma et al., 2015b), the evaluation and differentiation of mRNA cleavage and translational inhibition still requires more clarification for better application. Knowing that such synthetic nucleic acids generate a single miRNA with the ability to silence a specific target gene, all the potential unwanted target genes can be predicted and avoided during the experimental design stages (Schwab et al., 2010).

The ability of engineered decoys to modulate miRNA regulatory networks through modification of miRNA activity is also an approach for achieving a desired outcome (Ivashuta et al., 2011). miRNA decoys, endogenous RNAs that can negatively regulate miRNA activity, are a flexible and robust tool to understand the function of miRNA families, as well as for targeted engineering of gene expression in plants (Banks et al., 2012). This approach offers the possibility to alter the sequence of the miRNA decoy sites which attenuates miRNA inactivation, by allowing for fine regulation of native miRNA targets and the production of a desirable range of plant phenotypes. However, one of the challenges in successfully utilizing this approach for crop improvement is the ability to achieve a desirable level of miRNA inactivation to avoid off-type effects which can result in a false positive output. Also, the interaction between miRNA and the miRNA decoy, as well as the molecular complex that may form during this interaction, is not known and can lead to miRNA destabilization in plants. Therefore, for the practical benefit of this approach in crops, further clarification is needed on the mechanism underlying decoy-associated miRNA turnover and the contribution of decoys to global regulation of gene expression in plants.

With the realization of the potential of miRNA manipulation as a plausible tool in plant crop improvement, it is also important to be aware that such genetic modification approaches can lead to unintended side effects (Kamthan et al., 2015). If the expression of a miRNA or the target gene is altered, it can potentially result in undesirable pleiotropic changes in plant development and morphology. Therefore, it is imperative to understand the mechanisms of miRNA regulation of plant growth and development or plant responses to various abiotic and biotic stresses. This will facilitate the design of suitable strategies resulting in the desired traits but with minimum trade-offs in the modified crops.

## Author contributions

AD, NS, KN, and ID conceived and designed the review. All authors contributed to writing and editing the manuscript.

### Conflict of interest statement

The authors declare that the research was conducted in the absence of any commercial or financial relationships that could be construed as a potential conflict of interest.
